# Tissue Remodeling for a More Homogenous Ablation in Transoral Outlet Reduction Using Suturing and Noncontact Argon Plasma Coagulation

**DOI:** 10.14309/crj.0000000000001631

**Published:** 2025-03-06

**Authors:** Leandro Sierra, Arjun Chatterjee, Akash Khurana, Renan Prado, Roma Patel, Stephen A. Firkins, Roberto Simons-Linares

**Affiliations:** 1 Department of Internal Medicine, Cleveland Clinic, Cleveland, OH; 2 Department of Gastroenterology and Hepatology, Digestive Disease Institute, Cleveland Clinic, Cleveland, OH

**Keywords:** argon-plasma coagulation, endoscopic gastric revision, transoral outlet reduction, homogenous ablation, Roux-en-Y gastric bypass

## Abstract

The study is the first to assess the argon-plasma coagulation ArC Smart linear beam for mucosal ablation in transoral outlet reduction for weight regain after Roux-en-Y gastric bypass. The noncontact design of the linear beam can enhance tissue healing and remodeling of gastrojejunal anastomosis by causing a uniform scarring with minimal tissue injury. We followed patients for 45 days, during which none experienced gastrointestinal bleeding, stenosis, or ulcers, reporting 9.8% average of total body weight loss. The ArC Smart beam may offer a safer, more effective alternative to traditional argon-plasma coagulation for transoral outlet reduction, with better reduction in weight loss, although comparative studies are needed.

## INTRODUCTION

Argon-plasma coagulation (APC) is a well-established ablation method in gastrointestinal endoscopy and has been proven to be safe and effective.^1^ One notable use is for transoral outlet reduction (TORe), an endoscopic procedure aimed at reducing the size of the gastrojejunal anastomosis (GJA). This is typically performed when a patient experiences weight regain after a Roux-en-Y gastric bypass (RYGB), often to address issues like gastrogastric fistula and GJA dilation.^1^

An important step of the TORe procedure is ablating the GJA rim to promote effective fibrosis and scarring. The most common electrosurgical generators for this purpose are the Genii GI4000, ERBE ICC200/APC300, and ERBE VIO 300D/APC2.^2^ For performing the ablations, these ones produce a beam of constant length, where not the length, but only the intensity changes with power adjustments. In other words, increasing the power enhances the beam's intensity without altering its length.

One of the limitations of traditional APC is that its constant-length beam does not guarantee a noncontact effect.^3^ This fixed beam can cause deep tissue injury, including damage to the muscularis propria, potentially leading to mucosal perforation and significant tissue damage.^3,4^ In addition, traditional APC carries an increased risk of stenosis and presents a technical challenge for operators, who must carefully adjust power settings based on the patient.^3,4^

These limitations are intended to be addressed by adopting a noncontact approach between the APC device and the mucosa. This approach aims to ensure homogeneous tissue coagulation with a reduced risk of submucosal or serosal damage and to minimize the likelihood of tissue adhering to the argon probe, thereby lowering the risk of rebleeding.^4^

To take advantage from all the noncontact benefits, the ArC Smart linear beam has been created. This is a new technology that increases the beam length in a linear relationship with power increase. This way, increasing the desired “noncontact” approach and causing less depth of tissue injury.

The aim of the study is to examine the functionality of ArC Smart linear beam in mucosal ablation and to investigate its impact in tissue adaptation and scarring.

## CASE REPORT

### Device technical overview

Similar to traditional APC technology, the ArC Smart linear beam utilizes inert argon gas flowing through a hollow, flexible catheter.^4,5^ When sufficient voltage is applied between the probe's electrode and the tissue, ionization occurs.^4,5^

In the ArC Smart linear beam, the distance between the probe and the tissue, as well as the impedance of the total monopolar circuit, play a critical role.^5^ Once ionized, the gas plasma conducts high-frequency energy across the gap to the tissue, creating the desired thermal effect.^5^ As long as a sufficient argon cloud is present, the argon gas flow rate has minimal impact on the depth of tissue injury.^5^ The system adjusts the beam length in a linear relationship to the gap being filled and the power delivered, ensuring the desired noncontact effect between the probe and the tissue (Video 1).

### Procedure implementation

TORe using a modified-length beam (ArC Smart linear beam) was performed by Dr. Roberto Simons Linares in the endoscopic unit. Three patients, who were fully informed of potential risks and provided consent, underwent the procedure. All were hospitalized beforehand and received general anesthesia with noninvasive monitoring. Periprocedural antibiotics, including intravenous ciprofloxacin (500 mg twice daily) and metronidazole (500 mg 3 times daily), were administered for 3 days.

**Figure 1.**
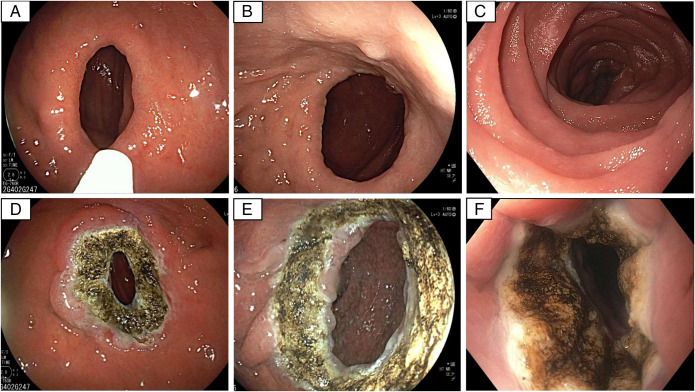
(A) Gastrointestinal outlet pre-ArC linear beam ablation for patient A. (B) Gastrointestinal outlet pre-ArC linear beam ablation for patient B. (C) Gastrointestinal outlet pre-Arc linear beam ablation for patient C. (D) Gastrointestinal outlet post-ArC linear beam ablation for patient A. (E) Gastrointestinal outlet post-ArC linear beam ablation for patient B. (F) Gastrointestina outlet post-ArC linear bean ablation for patient C.

In each patient, the GJA outlet was seen to be dilated from 25 mm to 40 mm in diameter (Figure 1). For the TORe revision, first the margin of the GJA was treated with APC, causing a homogeneous scarring (Figure 1). For all of the 3 patients, the APC was performed at a flow of 0.8 L/min, with a potency of 70 Watts. A suturing device (Overstitch, Boston Scientific, Marlborough, MA) was then used to do a purse-string pattern into the GJA anastomosis surrounding tissue. The suture was then tightened and secured over a 6-mm bougie. After the procedure, a diagnostic endoscope was used to evaluate the anatomy, without noticing any signs of mucosal injury. Finally, 2 vials of PuraStat (3D Matrix Europe SAS, Caluire-et-Cuire, France) were applied to the sutures to facilitate hemostasis.

All patients experienced smooth recoveries, with no instances of bleeding from the treated lesion or any infection. They resumed oral intake the day after treatment, beginning with a liquid diet. Upon discharge, patients were provided with proton pump inhibitors and sucralfate for a duration of 6 weeks, along with recommendations for diet and lifestyle changes.

Notably, patient C presented to the emergency department 10 days postprocedure due to a new-onset of severe nausea. This was attributed to be caused by her failure to take her medications as directed. She also reported feeling “isolated” due to her liquid diet. New recommendations for medication adherence were given, and the patient recovered uneventfully. Patients A and B experienced intermittent nausea but did not report vomiting or bloating (Table 1).

**Table 1.** Characteristics of the patients

Patient	A	B	C
Sex	F	F	F
Age at the time of TORe, yr	49	51	81
GJA outlet in mm	35	25	40
Pre-RYGB weight in lbs (BMI)	359 (56.1)	297 (51)	220 (39.9)
Post-RYGB nadir in lbs (BMI)	284 (44.4)	138 (23.7)	175 (31.7)
Pre-TORe weight in lbs (BMI)	288 (45)	186 (33)	182 (33)
6-wk post-TORe weight in lbs (BMI)	263 (41.1)	164 (29.1)	168 (32.7)
TBWL 6 weeks pre- and post-TORe, %	8.7	11.8	7.8
Time from RYGB to TORe revision, yr	5	5	16

BMI, body mass index; F, female; GJA, gastrojejunal anastomosis; RYGB, Roux-en-Y gastric bypass; TBWL, total body weight loss; TORe, transoral outlet reduction.

As demonstrated in Table 1, all the patients were female. The first and the second patient had similar age, 49 and 51 years, and both underwent TORe exactly 5 years after RYGB. The third patient was 81 years old and was having the revision procedure being performed 16 years post-RYGB (Table 1).

All 3 procedures took <20 minutes, leaving a homogeneous scar around the ring (Figure 1). GJA outlet size openings varied in our 3 patients, which can have an impact in the uniformity of the ablated tissue. Before endoscopic gastric revision, patient A had the highest BMI at 43 kg/m^2^, whereas the other 2 had similar BMIs before TORe at 33 kg/m^2^. All our patients lost weight within a month after TORe procedure, with the second having the highest total body weight loss at 9.6%. The average weight loss among the patients was 9.8% in 6 weeks (Video 1).

**Video 1** Implementation of the ArC Smart TM linear beam for mucosal ablation during Transoral Outlet Reduction post Roux-en-Y gastric bypass due to gastro-jejunal anastomosis outlet dilation.

## DISCUSSION

This is an innovative case series that uses a modified-length beam, the ArC Smart linear beam, increasing the noncontact time with the GJA mucosa, during the TORe procedure. Standardization of ablation technique for TORe procedure is still to be elucidated; however, an optimal ablation technique for TORe is necessary and could be complemented by other steps, such as full-thickness suturing and the application of hemostatic gels or healing-enhancing gels like PuraStat (3D Matrix Europe SAS).

Several techniques for TORe have been described in the literature, including APC alone (ie, APC-TORe procedure), full-thickness suturing combined with APC (ie, Suturing-TORe procedure), and endoscopic submucosal dissection (ESD) (ie, ESD-TORe procedure).^5–7^ However, procedures that involve APC usually necessitate a consistent and homogenous beam length to achieve the desired depth of ablation (mucosal and submucosal layers) and avoid complications (bleeding, perforation, postprocedure pain, nausea, or suboptimal healing and remodeling that can lead to procedure failure). As indicated in our hypothesis, this can result in less uniform scarring, higher risk of adverse side effects, with mucosal perforation or bleeding, and less total weight loss (TWL).

The mechanism of perforation during APC remains unclear, but it is thought to occur due to excessive or overablation in the same area, or also when the active argon probe contacts with the tissue for a prolong time, causing deeper thermal injury.^4,5^ This injury allows the argon gas to infiltrate the submucosa, leading to pneumatosis or extraintestinal gas accumulation.^5^ Other potential adverse events that could be mitigated by the noncontact approach include esophageal erosions, which, although less severe, can occur with traditional APC or submucosal erosion.^4^

A noncontact approach not only reduces complications but may also lead to more effective GJA ablation and improved weight loss outcomes. Dolan et al reported a TWL of 11.8% at 5 years post-TORe.^8^ Surprisingly, in our 6-week follow-up, the average TWL was 9.4%, approaching that 5-year figure in a much shorter time. This early result is comparable to the 8.5%–10.2% TWL seen in 1-year follow-ups from prior meta-analysis studies.^9^ We believe that better mucosal healing could be contributing to this enhanced weight loss, less postprocedure complications, although more research still has to be done.

It is crucial to consider patient-specific factors that may have influenced the results. Although 2 patients had similar BMIs, 1 had a significantly higher BMI, which may have skewed the average outcomes. Age is another important factor, as the oldest patient showed the smallest BMI reduction. This aligns with the expected trend of BMI increasing with age, particularly in postmenopausal women.^10^

There is an imperative need to evaluate our early findings in a larger prospective study, as they demonstrate apparent advantages over the traditional APC.^8,9^ Notably, the ArC Smart linear beam appears less aggressive than the traditional constant-length APC beam. Its increased power minimally impacts mucosal depth while ensuring effective and safe mucosal scarring with more homogeneous ablation. Furthermore, it achieved comparable weight loss in 6 weeks to studies reporting similar results over 6 months. This suggests that the ArC Smart linear beam in TORe may result in faster and greater overall weight loss. Additional benefits may include improved operator confidence, leading to faster procedures and a shorter learning curve compared to traditional models.

It is crucial to design new prospective studies comparing the different APC techniques as well as the different ablation settings of APC to validate its effectiveness in routine practice. This case series raises awareness of the different ablation modalities (devices, settings, and techniques) that still need to be defined and standardized for TORe procedure.

## DISCLOSURES

Author contributions: L. Sierra: conceptualization, project administration, writing the original draft, review, and editing; A. Chatterjee: writing the original draft, visualization, review, and editing; A. Khurana: writing the original draft, review, and editing; R. Prado: writing the original draft, review, and editing; R. Patel: visualization and editing; R. Simones-Linares: supervision, conceptualization, methodology, validation, and editing. All authors have read and agreed to the published version of the manuscript. L. Sierra is the article guarantor.

Financial disclosure: None to report.

Informed consent was obtained for this case report.
